# Global crop output and irrigation water requirements under a changing climate

**DOI:** 10.1016/j.heliyon.2019.e01266

**Published:** 2019-03-02

**Authors:** Victor Nechifor, Matthew Winning

**Affiliations:** UCL Institute for Sustainable Resources, 14 Upper Woburn Place, WC1H 0NN London, United Kingdom

**Keywords:** Environmental science, Economics, Agriculture

## Abstract

The anthropogenic increases in CO_2_ atmospheric concentrations are expected to lead to multiple and possibly opposing effects on crop performance with important implications for crop water productivity. The study integrates the global responses to mounting concentrations at three levels – climatic, cropping and economic – to determine the deviations in crop production and irrigation water requirements from a 'no climate change' socioeconomic development storyline. The biophysical effects are considered comprehensively for eight crop classes by taking into account alterations both to rainfed and irrigation yields, and to irrigation water intensities. These changes in crop growing conditions are explored in the 2004–2050 timeframe across two concentrations pathways (RCP2.6 and RCP 8.5) with the inclusion of the CO_2_ fertilisation effect. The economic responses are determined through a global water CGE model (RESCU-Water) comprising a bottom-up representation of crop systems. Changes in climatic conditions reduce crop output and depress the global water demand for irrigated crops in spite of an increase in irrigation water intensities. Discrepancies in crop production impacts between tropical and temperate regions increase with CO_2_ concentration levels. Embedding CO_2_ fertilisation more than offsets these adverse effects by determining a net increase in crop production and a reduction in irrigation water requirements at a regional level. The resulting water savings potential, even in the lower concentrations scenario (RCP2.6), warrant more research with the aim of reducing the different classes of uncertainty regarding the effects of CO_2_ fertilisation.

## 1 Introduction

Anthropogenic climate change is expected to have a significant impact on agricultural output (Porter et al., 2014). The relationship between increases in concentrations of greenhouse gases, in particular CO_2_, and crop growth is composed of multiple and possibly opposing effects (Gornall et al., 2010). On an annual basis, crop yields would be affected directly by changes in mean climatic conditions (temperature, precipitation, length of growing seasons) but also indirectly through the fertilisation effect of CO_2_ due to the enhancement of photosynthesis of C3 plants.^1^

From a water use perspective, CO_2_ fertilisation (referred to in this article as CF) may also lead to higher crop water efficiencies through a lower transpiration at the leaf level (Wullschleger et al., 2002) and could thus appreciably alter water requirements for crop production (Betts et al., 2007). At the same time, changes in precipitation patterns would modify the natural soil water balance. As some areas are expected to have an increase in annual precipitation levels, the intensity of blue water usage on irrigated land to compensate for any soil moisture deficiencies for optimal crop growth could thus be reduced (Döll, 2002; Fader et al., 2010; Gerten et al., 2011).

Crop demand growth due to socioeconomic development will lead to increases in irrigation water requirements in most regions (Nelson et al., 2010; Alexandratos and Bruinsma, 2012; Nechifor and Winning, 2017). Therefore, the incidence of climate change will become more pronounced at a time with growing pressure on freshwater resources coming from crop production. This conjunction of socioeconomic and climatic drivers of freshwater use indicate the importance of considering the implications of climate change on freshwater demand when analysing the state of future water scarcity.

There are now several assessments on global changes in crop water productivity using climatic and crop modelling techniques (Rosenzweig et al., 2014), however, the conversion of these biophysical alterations into absolute water requirements for irrigation has not been conducted by accounting for demand and supply interactions in the global crop markets. Many economic models, able to capture these microeconomic responses, have indeed been used to determine the effects of changes in mean climatic conditions over crop output (Nelson et al., 2014; Wiebe et al., 2015; Fischer et al., 2005; Palatnik and Roson, 2011; Darwin et al., 1995; Calzadilla et al., 2013; Taheripour et al., 2013a,b). However, from a natural resource use perspective, most analyses have focused on the land-use change dimension (Nelson et al., 2014; Taheripour et al., 2013a,b; Gurgel et al., 2007; Lampe et al., 2014) and less on the implications for blue water requirements in crop production with a comprehensive inclusion of factors influencing irrigation water intensities.

Furthermore, the effect of CF has not always been taken into account (Porter et al., 2014) despite its potentially non-negligible implications on crop performance (Müller and Robertson, 2014) and crop water intensities (Deryng et al., 2016). The main deterrent for not considering CF in economic models is the uncertainty of the extent to which this effect will materialise given its non-linear interactions with other factors – climatic (temperature, humidity) and other GHG concentrations (ozone precursors). Whilst laboratory trials have so far been conducive to the enhancing effect of higher CO_2_ concentrations over yields, large-scale experiments are still under development. Nevertheless, this effect with regard to yields and crop water productivity is currently considered in a growing number of global crop models (Warszawski et al., 2014).

The present study determines the changes to future irrigation water demand by integrating the responses of rising CO_2_ concentrations at three different levels – climatic, crop-level and economic. Whilst the data for the first two levels is currently produced by established global circulation and crop models and was recently made available through the ISI-MIP project (Warszawski et al., 2014), the changes in regional water demand as a function of crop performance calculated through global economic modelling is still under-represented (see Nechifor and Winning, 2017 for an overview). The analysis thus adds an important component to the assessment of future water through a bottom-up consideration of changes in global crop markets and in the underlying irrigation water requirements. The assessment covers the 2004–2050 timeframe and explores the alterations to crop performance across two CO_2_ concentrations pathways (RCP2.6 and RCP8.5) and two CF variants (with and without CF). For this analysis, the additional effects of climate mitigation policies on crop output and water requirements are not considered. Bioenergy can prove to have a significant role in the future abatement of CO_2_ emissions, notably for low-concentration pathways such as RCP2.6. Nevertheless, at this stage, there are still large uncertainties regarding the extent to which bioenergy will be adopted, and also regarding the land- and water-use implications of a large-scale adoption of purpose-grown crops.

The study is structured as follows. After this introduction, Section 2 presents the response types to mounting CO_2_ concentrations considered in the present assessment. As multi-model biophysical data is already available at high spatial detail, the emphasis is placed on the methods to integrate this information into a global economic modelling framework. Section 3 compares the aggregated yield and water intensity changes across the two CF variants. The crop output and water requirements outcomes including the economic response to changes in yields are then presented. The main drivers of changes in crop water productivity (CWP) are also explored through a decomposition analysis. Section 4 discusses the significance of the results and the limitations of the current study. Section 5 concludes.

## 2 Methods

### 2.1 Climatic and crop-level responses

Climatic changes coming from higher CO_2_ concentrations are included through alterations in daily temperature, precipitation patterns and cloud cover and are determined through global circulation models (GCMs). Using this climatic data, crop modelling results from the LPJmL (Lund-Potsdam-Jena managed Land) model (Bondeau et al., 2007) are then employed to explore the indirect effect of CO_2_ concentrations on yields and crop water intensities through the CF effect. The combined climatic and crop-level responses are considered through two biophysical parameters – *yields* differentiated by crop class and by growing method (rainfed and irrigated), and *irrigation water intensities* differentiated by irrigated crop class. The irrigation water intensities included in LPJmL represent the compensation for soil water deficiencies required for optimal plant development, and do not include any potential water use constraints coming from a limited availability of blue water. Changes in these two parameter sets are determined using the LPJmL crop model output with data published on the ISI-MIP FastTrack platform.^2^ The choice of LPJmL among all crop models participating in the inter-comparison project was based on the largest coverage of scenarios across crop classes, RCPs and CF variants. The yields and water intensity data are calculated at a global level on an annual basis using a 0.5° spatial resolution and are based on daily information for CO_2_ concentrations, temperature, precipitation and radiation.

Eleven crop types included in the LPJmL simulations for ISI-MIP were selected to determine the changes in two biophysical parameters of the eight crop types represented in the economic model detailed below (see Table 1). Whilst the mapping is one-to-one for some crop types (wheat, rice and soy), other crop classes require more detail due to differences between the representative crops of the main agro-ecological zones. For instance, for the *cane & beet* crop class used in the economic model, changes were considered for sugar beet in temperate regions and for sugarcane in tropical regions.

**Table 1:** RESCU-Water – LPJmL crop mapping.

Application	RESCU-Water crop	LPJmL crop
Wheat	Wheat	Wheat
Paddy rice	Rice	Rice
Other grains tropical	Other grains	Millet
Other grains temperate	Other grains	Maize
Veg & fruits tropical	Veg & fruits	Cassava
Veg & fruits temperate	Veg & fruits	Field Pea
Cane and beet temperate	Cane & beet	Sugar Beet
Cane and beet tropical	Cane & beet	Sugarcane
Oil seeds	Oil seeds	Soy
Plant fibers	Fiber plants	Managed Grass
Other crops	Other crops	Weighted average of the above

Source: own construction.

The LPJmL outputs are expressed in values per hectare and reflect the raster point growing conditions without taking into account actual harvested areas. Therefore, similarly to Villoria et al. (2016), changes at a regional level are determined as averages by factoring in cropping maps from MIRCA2000 (Portmann et al., 2010) aggregated to the RESCU-Water regions.

Thus, yields per LPJmL crop class *crop*, growing method *m* and region *r* are calculated using the following weighted average:

(1) $$Y_{r,crop,m}^{t}=\frac{\sum_{p_{r}}area_{crop,\;p_{r},m}*yield_{crop,\;p_{r},m}^{t}}{\sum_{p_{r}}prod_{crop,\;p_{r},m}}$$

where *p*_*r*_ represent all the raster points within each RESCU-Water region *r*. Harvested area for each crop LPJmL crop class $area_{crop,\;p_{r}}$ is taken from the MIRCA2000 dataset whilst yield data $yield_{crop,\;p_{r}}^{t}$ is determined by LPJmL.

Water intensities are calculated by tracking the changes of the LPJmL *potential irrigation water withdrawal* $pirrww_{irc,\;p_{r},m}^{t}$ variable of each crop type and for each region. The regional aggregation is done in a similar fashion as for yields:

(2) $$PIRRWW_{r,crop}^{t}=\frac{\sum_{p_{r}}area_{crop,\;p_{r}}*pirrww_{crop,\;p_{r}}^{t}}{\sum_{p_{r}}prod_{crop,\;p_{r}}}$$

To address the issue of climate change incidence uncertainty, the crop model data were considered in relation to the climate data of three GCMs (MIROC-ESM-CHEM, HadGEM-ES, IPSL-CM5). Also, to filter the effects of climate variability on model results, a two-sided 21-year moving average was used for both parameter sets.

### 2.2 Economic responses

**Fig. 1:**
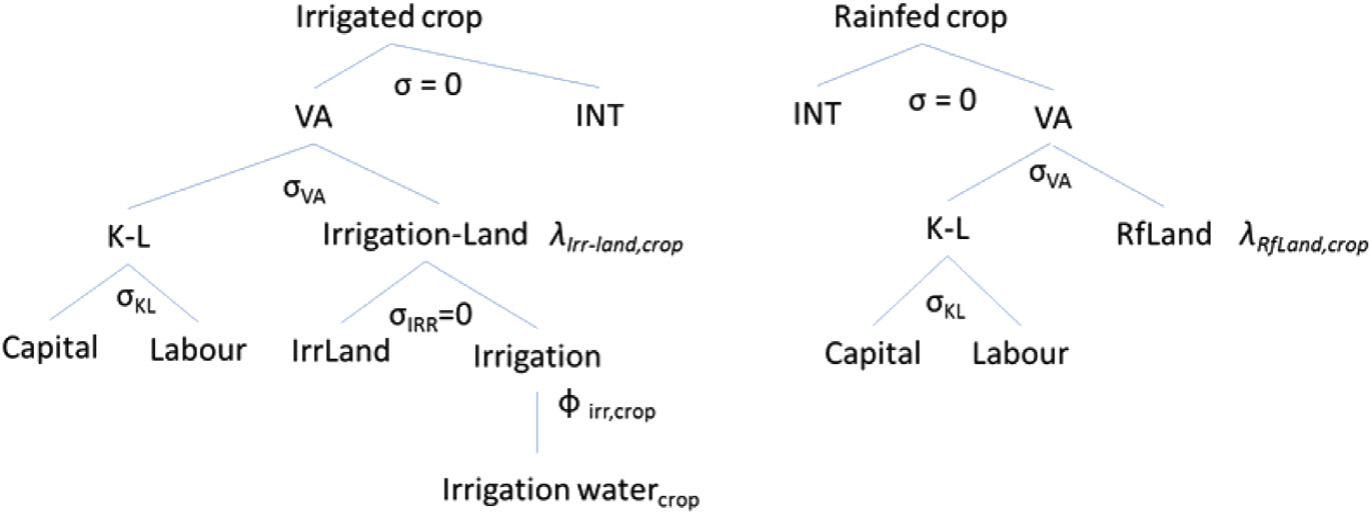
RESCU-Water irrigated and rainfed crop production functions.

The economic responses are determined using the RESCU-Water (Resources CGE UCL, see Nechifor and Winning (2017) for the full model description), a computable general equilibrium (CGE) model which comprises a bottom-up representation of global crop systems structured around 20 world regions. The model includes eight crop classes (*rice*, *wheat*, *other grains*, *veg & fruits*, *fiber plants*, *cane & beet*, *oil seeds* and *other crops*) and specifies irrigated and rainfed production of each class as distinct economic activities (Fig. 1). This advanced specification allows for a wider consideration of the impacts of climatic and CF changes on crop yields and thus captures the substitution effects between crops and growing methods based on market price signals. The model is calibrated using the GTAP 9 database (Aguiar et al., 2016) and runs recursively at yearly time steps. Technological change is introduced through increases in labour and agricultural land productivities over time. Labour and capital availability for the productive sectors in each region are updated to follow the evolution of investment and population respectively. On the consumption side, the model implements a Stone-Geary demand system which enables the consideration of a subsistence component of household demand across 16 consumer commodities. Bilateral international trade is captured by employing the Armington assumption (Armington, 1969) through which the domestic and imported varieties of a commodity are introduced as imperfect substitutes.

The RESCU-Water model baseline for 2004–2050 is constructed using the SSP2 “middle of the road” storyline for economic and demographic evolution (O'Neill et al., 2014). For this development pathway, the global population would reach 8.9 billion by 2050 while the global GDP would increase 3.2 times from 2004 levels. The GDP and population projections used in the baseline are obtained from the IIASA SSP database and do not assume any feedback of climate change socioeconomic development. Population growth impacts the model results by changes in regional labour supply, and by changes to subsistence consumption updated yearly to reflect a growing demand for basic food demand. The GDP targets are achieved in the model through increases in capital availability driven by investment rates in accordance with SSP2 (Fouré et al., 2013) and through labour-augmenting technical progress.

In the model simulations, the yield alteration information obtained from the aggregation of the LPJmL data in equation *(1)* is implemented as factor productivity changes to the land-related inputs of both irrigated (*λ*_*Irr-Land,crop*_) and rainfed production (*λ*_*RfLand,crop*_). The baseline values for *λ* reflect the expected technological advancements as calculated by the IMPACT model (Nelson et al., 2010) and correspond to conditions of perfect climate change mitigation. The economic responses thus translate into alterations to the regional crop production mix and consider the use of irrigation as a potential adaptation measure to climate change.

The calculation of water demand for irrigation is enabled through the inclusion of irrigation as a distinct factor of production for irrigated crops (see irrigation and rainfed production technologies in Fig. 1). The availability of *Irrigation water* is endogenised and can expand or contract based on changes in crop market conditions and on arable land conversion from rainfed to irrigable. Water use by crop class is tied to the allocation of the *Irrigation water* factor across the RESCU-Water crop classes and is determined through crop-specific irrigation water intensities $\phi_{irr,crop}$. Crop water intensities refer to the required amount of blue water (inclusive of the irrigation efficiency factor) per dollar of crop output. These intensities are calculated for each crop at a regional level based on spatially-detailed global irrigation practices as determined by GCWM (Global Crop Water Model – Siebert and Döll, 2010) in combination with the changes in irrigation water requirements determined in equation *(2)*.

Yield and water intensity changes are considered for both CF variants (with and without CF) in each RCP scenario. Changes in the two parameter sets corresponding to the climate data of each of the three GCMs considered are included through separate RESCU-Water model runs. The results for the main scenarios are reported, however, as averages across the three circulation models.

The model results are compared across the lowest (RCP2.6) and highest (RCP8.5) radiative forcing scenarios. CO_2_ concentrations between these two RCPs start to significantly diverge from 2025 and lead to a 100 ppm span by 2050. Therefore, when getting closer to the end of the simulation period, in addition to changes in climatic conditions, the size of the CF effect becomes increasingly sensitive to the concentration pathway choice. Emission patterns for the SSP2 socioeconomic scenario are projected in van Vuuren et al. (2014) to determine radioactive forcing levels in the RCP6.0-RCP8.5 range. Nevertheless, the lower RCP2.6 is taken into account in this study to explore the outcomes of climate action on crop output and irrigation water requirements.

It should be noted that only yields act as shock variables in the RESCU-Water simulations. These alterations to crop performance determine a new equilibrium point across crop markets through a change in the cost structure – a degradation of yields leads to higher costs of production due to higher land input requirements. The corresponding change in land costs lead to an overall crop price effect with impacts over demand but also to a substitution of land and irrigation in relation to the other inputs of crop production. In contrast, the water intensities indicate the levels of water required by the use of the *Irrigation water* factor across crops, but do not affect the supply and allocation of irrigation or cropland.

## 3 Results

### 3.1 Aggregated yields and irrigation water intensities

**Fig. 2:**
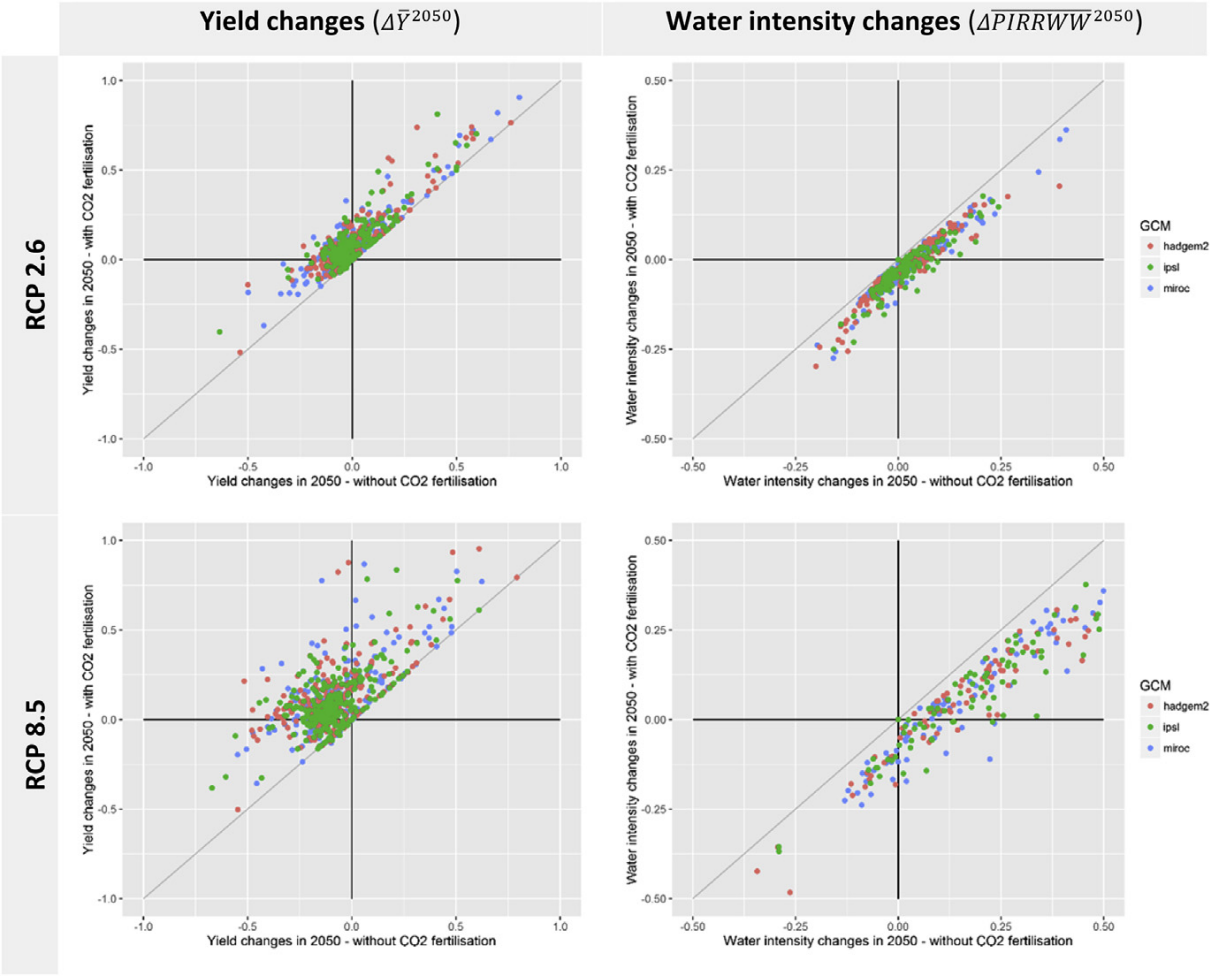
Comparison of yield and irrigation water intensity changes between CO_2_ fertilisation variants and across GCMs. Note: Each point represents one crop variety (rainfed and irrigated by crop class) within one RESCU-Water region – all modelled cases covered in this representation. Source: own construction.

The results of the crop data aggregation outlined above are presented in Fig. 2 for 2050 by comparing yield and water intensity changes at a regional level across the two CF variants. Without CF, yields are generally degraded (points left of the y-axis) and this tendency is amplified with the growth in CO_2_ concentrations (RCP8.5). Crop water intensities outcomes are mixed for RCP2.6 (points on both sides of the y-axis). However, a general increase in intensity is observed with RCP8.5.

With CF, yields obtain a net improvement (points above the x-axis) which is increasing with CO2 concentrations. At the same time, some crops (C4) are indifferent to the fertilisation effect in terms of yield changes (points on the diagonal). This indifference is not applicable to water intensities as the CF-induced water efficiency is found in both C3 and C4 crop types (all points are below the diagonal indicating lower water intensities *with CF* compared to *without CF* for all crops in all regions).

### 3.2 Global crop impacts

**Fig. 3:**
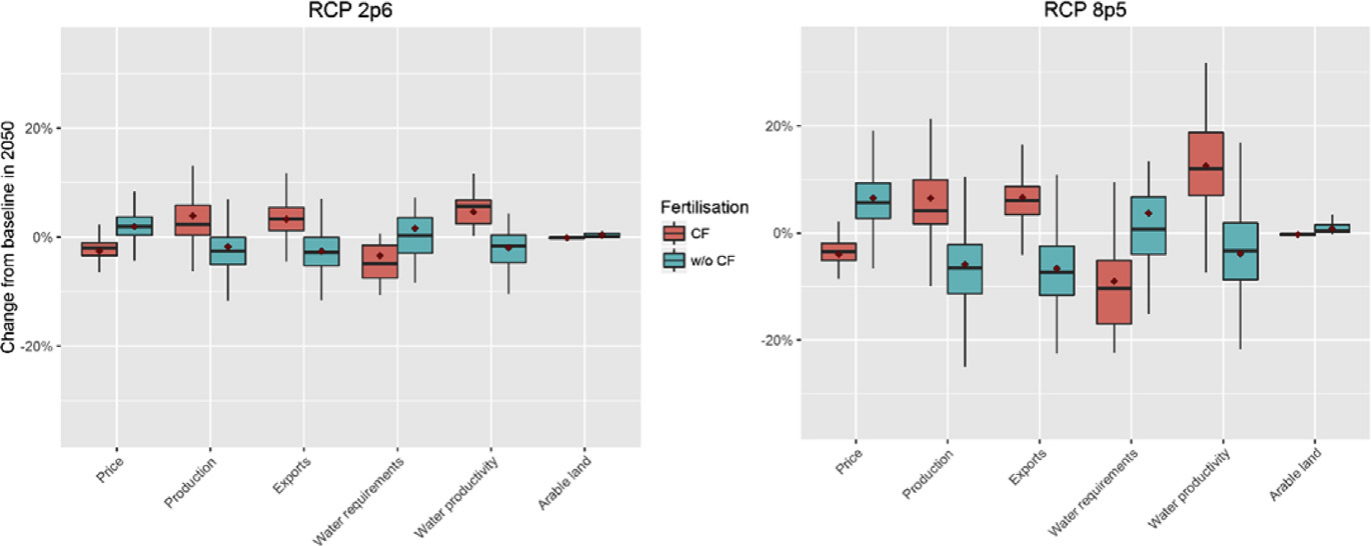
RCP 2.6 and RCP 8.5 changes in main crop variables (% change from 2050 baseline values). Note: the whiskers and boxes indicate the 5th, 25th, 50th, 75th and 95th percentiles. The diamonds represent the mean values. Source: own construction.

In 2050, changes in crop growing conditions have a visible impact over crop sectors even for the low emissions pathway RCP2.6. Deviations from the baseline and the variance of climate change incidence across regions increase with CO_2_ concentrations. Fig. 3 shows the changes relative to the baseline values in 2050 to the main crop market variables (price, output and exports) and resource use (water requirements and arable land). The boxplots illustrate the combined results across crop types and regions.

The cost effect of the yield evolution is noticeable through changes in crop market prices. The results show opposing impacts of the two CF variants. Whilst climate change increases prices and determines a reduction in crop output when CF is not considered, fertilisation more than offsets the loss of yields induced by climatic conditions, leading to an overall crop price decrease and a boost to crop production compared to the baseline. The size of international trade of crops measured through regional exports changes in the same direction as crop output.

The water productivity changes represented are endogenous to the economic model. These reflect the evolution of water intensities as calculated by the LPJmL model, but also include the alterations to the allocation of irrigation infrastructure and equipment across crop classes given the input substitution effect and changes in the crop production mix.

### 3.3 Changes in irrigation water requirements

**Fig. 4:**
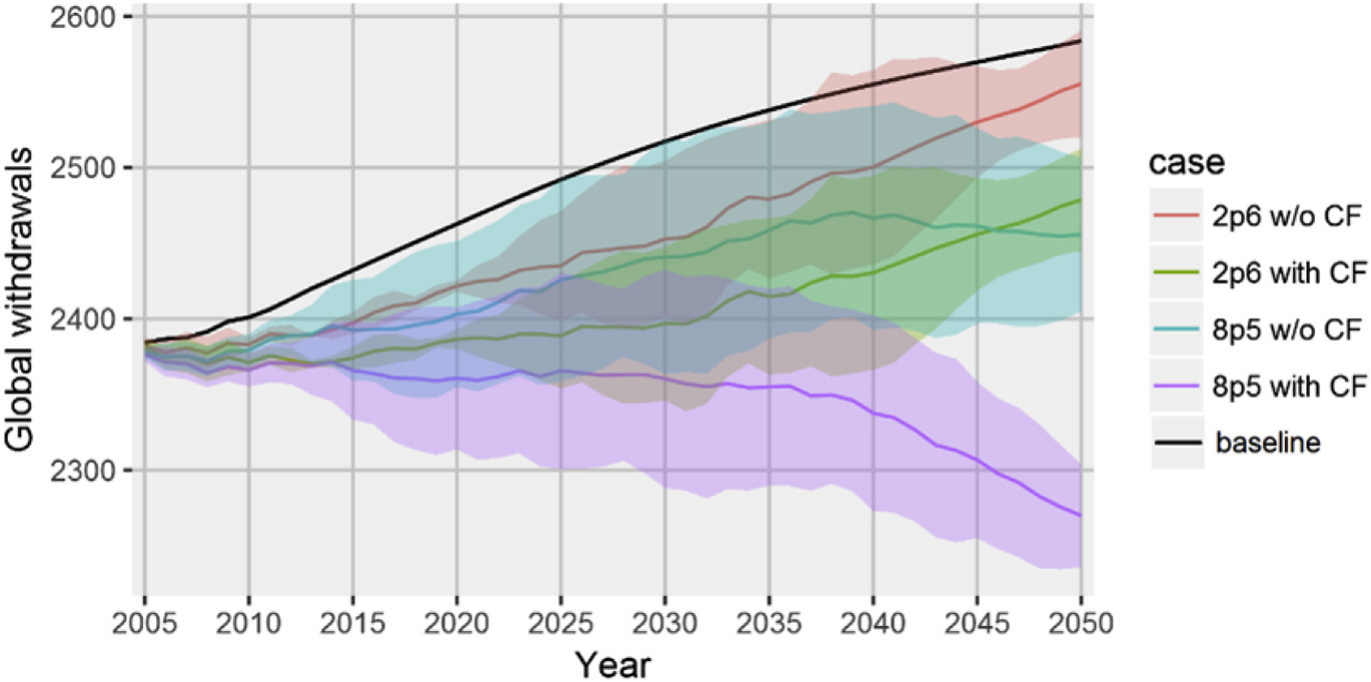
Global irrigation water withdrawals (in km^3^) by climate change scenario – 2005–2050. Note: the lines represent the mean values obtained across the three GCMs, the ribbons indicate the standard deviation. Source: own construction.

Global water requirements in irrigation decline with the increase in CO_2_ concentrations in both CF variants. Without the CF effect, requirements in 2050 are 1.1% and 5% lower for RCP2.6 and RCP8.5 respectively compared to the baseline (Fig. 4). This decline is primarily due to the overall decrease in crop production. However, part of this effect is counter-balanced by a reduction in irrigation water productivity in many regions. Despite the expansion in crop output, CF determines an even higher reduction in water requirements – 4.1% (RCP2.6) and 12.2% (RCP8.5).

The global changes in irrigation water requirements are concentrated in irrigation intensive regions (Tables 2 and 3). With CF, the increase in crop production determines a reduction in regional water requirements in all regions, except China, Northeast Asia and Northern Europe. In RCP8.5, the reductions in India account for more than a third of the world total. South Asia, the Middle East and Northern Africa are also important drivers in decreasing the global water demand.

**Table 2:** Changes in regional water requirements (in km3) by crop type and by CF variant – RCP 2.6.

Region	Overall		Wheat		Rice		Other grains		Veg & fruits		Fiber plants		Cane & beet		Oil seeds		Other crops	
	CF	w/o CF	CF	w/o CF	CF	w/o CF	CF	w/o CF	CF	w/o CF	CF	w/o CF	CF	w/o CF	CF	w/o CF	CF	w/o CF
Australia & NZ	0.0	0.3	(0.1)	(0.1)	(0.1)	(0.1)	(0.1)	(0.1)	(0.1)	(0.0)	0.4	0.5	0.1	0.0	0.0	0.0	(0.1)	0.0
Brazil	(0.7)	0.0	(0.0)	0.0	(0.2)	(0.1)	(0.0)	(0.0)	(0.2)	0.2	0.0	0.0	(0.1)	(0.2)	(0.0)	0.0	(0.1)	0.0
Sahel	(0.9)	0.1	(0.0)	(0.0)	(0.2)	0.6	(0.0)	(0.0)	(0.1)	(0.2)	0.0	0.0	(0.2)	(0.2)	0.0	0.0	(0.3)	(0.0)
Central Africa	(1.9)	1.4	(0.0)	(0.0)	(0.7)	1.4	0.4	0.4	(0.6)	(0.0)	(0.0)	(0.0)	(0.1)	(0.2)	0.0	0.0	(0.8)	(0.2)
Central Asia	(2.9)	0.4	(4.7)	(2.7)	(0.0)	(0.1)	(1.7)	(1.0)	(1.5)	(0.9)	5.1	5.2	(0.0)	(0.0)	(0.0)	0.0	(0.0)	(0.2)
China	33.4	43.2	(0.6)	1.2	21.8	21.4	6.6	9.0	1.9	5.7	1.5	2.0	(0.9)	(0.8)	3.0	4.6	0.1	0.1
Eurasia	(2.0)	(0.9)	(1.4)	(1.0)	(0.1)	(0.2)	(0.1)	0.2	0.0	0.3	0.0	0.3	0.0	0.1	(0.1)	(0.0)	(0.5)	(0.5)
India	(58.7)	(46.2)	(31.8)	(25.9)	8.8	6.0	(0.7)	(0.7)	(4.4)	1.6	(10.0)	(8.6)	(10.0)	(10.9)	(2.4)	(1.2)	(8.2)	(6.4)
Middle East	(15.2)	(4.7)	(6.1)	(5.8)	(0.3)	(0.3)	(0.0)	0.2	(7.2)	1.3	4.2	5.4	(0.5)	(0.7)	(0.8)	(0.6)	(4.6)	(4.2)
Northern Africa	(14.1)	(6.3)	(1.4)	(2.0)	(1.4)	(1.2)	(4.5)	(4.2)	(3.4)	(0.5)	3.2	3.5	(1.6)	(1.6)	(0.2)	(0.1)	(4.8)	(0.2)
Northeast Asia	1.0	1.6	(0.0)	0.0	0.8	1.3	0.0	0.0	(0.0)	0.0	0.0	0.0	(0.0)	0.0	0.2	0.2	0.1	0.1
Northern Europe	(0.0)	0.1	(0.0)	(0.0)	0.0	0.0	(0.0)	0.0	0.0	0.1	0.0	0.0	(0.0)	0.0	0.0	0.0	(0.0)	0.0
North Latin Am	(1.9)	(0.9)	(0.8)	(2.2)	0.3	(0.6)	1.0	1.2	(2.3)	(0.1)	0.6	0.6	(0.7)	(1.1)	(0.2)	1.2	0.3	0.2
Canada	(0.2)	(0.2)	(0.0)	(0.1)	0.0	0.0	0.0	(0.0)	(0.0)	0.0	0.0	0.0	0.0	0.0	(0.1)	(0.1)	(0.0)	(0.1)
Southern Africa	(0.3)	0.1	(0.1)	(0.2)	0.0	0.0	0.3	0.5	0.1	0.3	(0.0)	0.0	(0.3)	(0.3)	0.0	0.0	(0.2)	(0.2)
South Asia	(22.7)	(16.7)	(7.9)	(8.1)	(1.5)	(1.1)	(1.0)	(0.4)	(4.9)	(1.8)	(2.3)	(2.0)	(2.8)	(2.3)	(0.1)	0.0	(2.1)	(1.1)
Southeast Asia	(7.4)	(0.6)	(2.2)	(1.2)	(5.4)	(4.2)	0.0	0.0	1.7	4.3	(0.0)	0.0	(1.1)	(0.4)	(0.0)	0.0	(0.4)	0.8
Southern Europe	0.5	4.8	(0.3)	(0.2)	(0.1)	(0.1)	1.1	1.4	(1.5)	1.3	1.6	1.8	0.0	0.1	(0.2)	0.0	(0.3)	0.6
South Latin Am	(0.4)	0.7	(0.1)	(0.1)	(0.1)	(0.2)	0.2	0.4	(0.2)	(0.1)	0.1	0.0	(0.5)	(0.5)	0.2	1.1	0.0	(0.1)
USA	(10.8)	(4.8)	(2.3)	(2.1)	(0.8)	(0.2)	(2.3)	(2.4)	(1.2)	0.3	0.7	1.9	(0.1)	(0.1)	(4.4)	(1.8)	(0.4)	(0.4)
**World**	(105.1)	(28.4)	(59.8)	(50.4)	20.6	22.5	(1.0)	4.7	(24.0)	11.9	5.2	10.6	(18.8)	(19.1)	(5.0)	3.4	(22.4)	(11.9)

Source: own calculation.

**Table 3:** Changes in regional water requirements (in km^3^) by crop type and by CF variant – RCP 8.5.

Region	Overall		Wheat		Rice		Other grains		Veg & fruits		Fiber plants		Cane & beet		Oil seeds		Other crops	
	CF	w/o CF	CF	w/o CF	CF	w/o CF	CF	w/o CF	CF	w/o CF	CF	w/o CF	CF	w/o CF	CF	w/o CF	CF	w/o CF
Australia & NZ	(0.4)	0.3	(0.1)	(0.1)	(0.2)	(0.2)	(0.1)	(0.0)	(0.3)	(0.0)	0.4	0.6	0.0	(0.1)			(0.1)	0.3
Brazil	(2.1)	(0.4)	(0.0)	0.0	(0.9)	(0.5)	(0.0)	(0.0)	(0.6)	0.3	0.0	0.0	(0.2)	(0.3)	(0.0)	0.1	(0.3)	0.1
Sahel	(1.9)	0.1	(0.0)	(0.1)	(0.8)	0.9	(0.0)	(0.0)	(0.0)	(0.2)	(0.0)	0.0	(0.4)	(0.4)			(0.7)	(0.0)
Central Africa	(5.4)	3.2	(0.0)	(0.0)	(2.5)	3.6	0.4	0.4	(1.2)	0.2	(0.1)	(0.1)	(0.2)	(0.2)	(0.0)	0.0	(1.8)	(0.7)
Central Asia	(4.0)	5.1	(5.8)	(0.9)	(0.5)	(0.6)	(3.2)	(1.6)	(2.9)	(1.3)	8.5	10.1	(0.1)	(0.0)	0.0	0.0	(0.1)	(0.5)
China	8.4	33.7	(7.8)	(4.2)	13.8	15.0	3.0	9.1	(2.4)	6.3	1.4	2.8	(1.6)	(1.2)	1.9	6.0	0.1	0.0
Eurasia	(3.7)	(1.0)	(2.0)	(1.2)	(0.2)	(0.3)	(0.5)	0.3	(0.2)	0.5	(0.1)	0.5	0.0	0.1	(0.1)	(0.0)	(0.7)	(0.7)
India	(144.3)	(119.7)	(70.0)	(55.7)	(10.6)	(16.2)	(0.5)	(0.5)	(6.0)	12.2	(11.2)	(8.7)	(34.1)	(43.5)	(1.5)	2.8	(10.3)	(9.9)
Middle East	(34.2)	(7.7)	(8.5)	(6.9)	(1.0)	(0.7)	(1.6)	(0.6)	(18.3)	0.3	3.3	6.4	(0.4)	(0.5)	(0.9)	(0.3)	(6.8)	(5.3)
Northern Africa	(28.5)	(11.3)	(1.7)	(2.8)	(2.9)	(2.4)	(7.6)	(7.3)	(6.8)	0.2	4.2	4.7	(2.4)	(2.3)	(0.3)	(0.1)	(10.7)	(1.2)
Northeast Asia	1.4	3.0	(0.0)	0.0	1.1	2.5	(0.0)	0.1	(0.0)	0.1			0.0	0.0	0.2	0.2	0.1	0.1
Northern Europe	0.1	0.5	(0.0)	0.0			0.0	0.0	0.0	0.3			0.0	0.1			(0.0)	0.1
North Latin Am	(5.8)	(1.8)	(0.9)	(4.1)	0.3	(1.6)	0.3	0.8	(4.2)	0.9	0.6	0.6	(1.5)	(2.0)	(0.4)	4.2	(0.1)	(0.6)
Canada	(0.3)	(0.2)	(0.1)	(0.1)			0.0	0.0	(0.1)	0.1					(0.1)	(0.0)	(0.1)	(0.1)
Southern Africa	(0.7)	0.3	(0.0)	(0.3)	(0.0)	(0.0)	0.6	1.1	(0.2)	0.3	(0.0)	0.0	(0.5)	(0.6)	0.0	0.1	(0.5)	(0.4)
South Asia	(49.4)	(34.6)	(15.9)	(16.8)	(7.5)	(6.4)	(2.6)	(0.9)	(8.2)	(0.8)	(2.6)	(1.0)	(8.0)	(6.7)	(0.1)	0.3	(4.4)	(2.3)
Southeast Asia	(17.9)	0.0	(4.3)	(2.1)	(10.9)	(5.3)	0.0	0.0	1.5	7.9	(0.0)	0.0	(3.0)	(1.9)	(0.2)	(0.1)	(1.0)	1.6
Southern Europe	(4.2)	6.3	(0.6)	(0.3)	(0.2)	(0.1)	0.9	1.8	(4.1)	2.3	1.4	1.7	0.0	0.1	(0.6)	(0.1)	(1.0)	0.9
South Latin Am	(1.8)	0.5	(0.2)	(0.2)	(0.2)	(0.5)	0.3	0.8	(0.6)	(0.2)	0.1	0.0	(0.7)	(0.7)	(0.4)	1.5	(0.0)	(0.3)
USA	(19.5)	(4.4)	(4.6)	(4.6)	(0.4)	1.3	(4.5)	(4.4)	(3.5)	0.0	1.0	4.7	(0.3)	(0.3)	(6.6)	(0.7)	(0.7)	(0.5)
**World**	**(314.1)**	**(128.0)**	**(122.6)**	**(100.6)**	**(23.6)**	**(11.7)**	**(15.3)**	**(1.0)**	**(58.0)**	**28.9**	**7.0**	**22.4**	**(53.4)**	**(60.5)**	**(9.1)**	**13.7**	**(39.0)**	**(19.4)**

Source: own calculation.

Without CF, the global reduction is largely driven by India through a significant drop in *rice* and *wheat* production (see Section 3.4), with decreases also found in a number of other regions (Middle East, Northern Africa, South Asia and USA). At the same time, just a few regions face a significant increase in irrigation water demand due to a higher reliance on irrigated crops. China has the largest expansion driven by lower water productivity leading to an increase in water demand for *rice*, *other grains*, *veg & fruits, oil seeds* and *fiber plants*. Other increases in regional withdrawals are determined by higher water requirements for *rice* in Central Africa and Northeast Asia, *fiber plants* in Central Asia and Southern Europe, and *other grains* in Southern Europe.

Across crops, the most significant decreases occur for *wheat* and *cane & beet* in both CF variants. At the same time, the largest contrasts are obtained for *veg & fruits* and *oil seeds* – a decrease in water requirements with CF and an increase without CF. These classes are also high-value crops which determine a re-allocation of irrigation away from other types when adapting to the changes in climatic conditions.

### 3.4 Crop-specific impacts

**Fig. 5:**
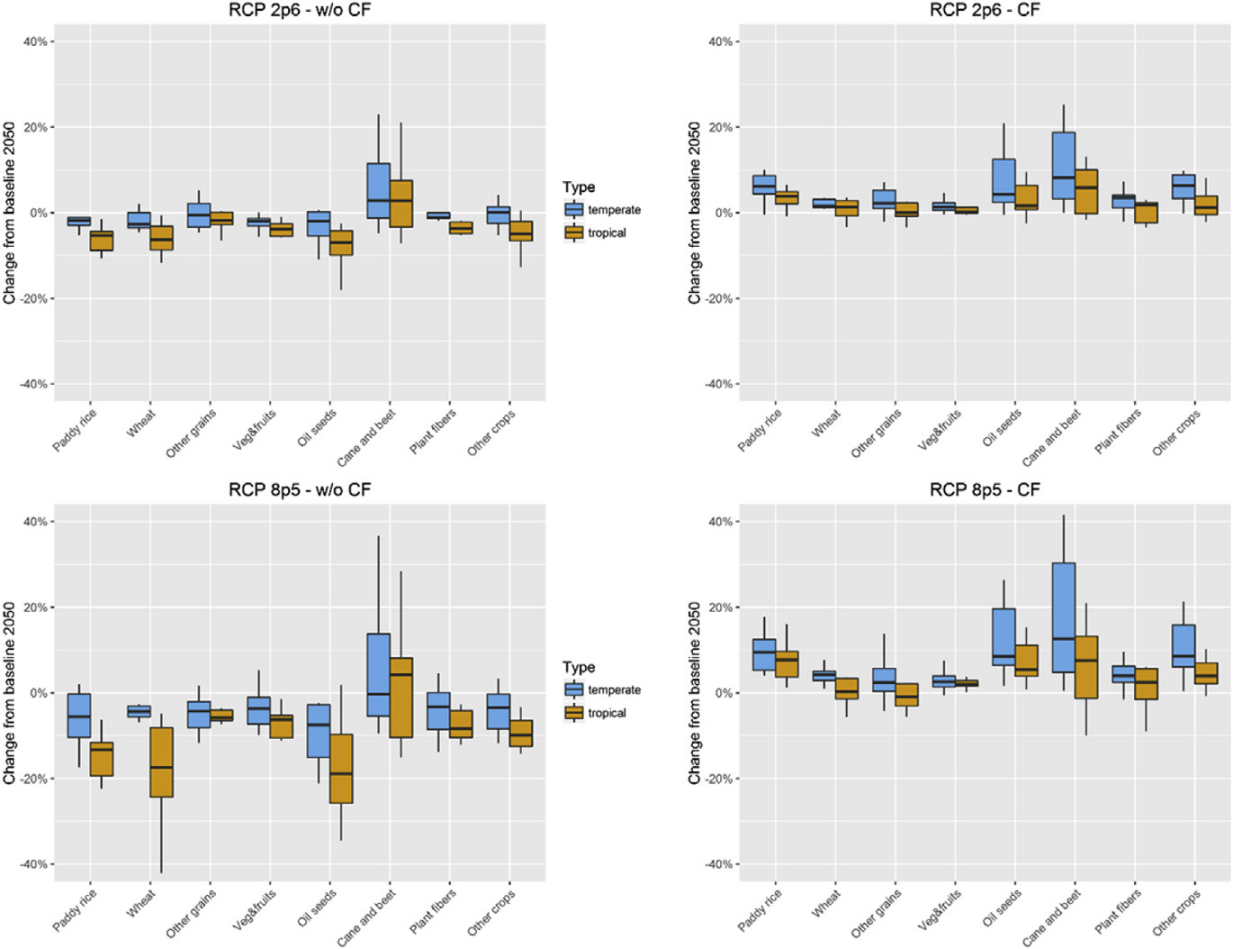
Crop output changes by RCP and by CO_2_ fertilisation variant. Source: own construction.

The incidence of climate change can be differentiated by grouping regions into their preponderant climate type (Fig. 5). Without CF, changes in climatic conditions alone have a stronger adverse impact over crop output in tropical regions. The *cane & beet* group is mostly positively affected by changes in climatic conditions. As a C4 crop, sugar cane is indifferent to CF, hence the fertilisation effect for *cane & beet* is more visible in temperate regions where sugar beets are grown predominantly. Although the total effect over output relative to the baseline remains stronger in temperate regions, CF plays an important role in correcting some of the distributional effects of climate change on crop output across regions – except for the *other grains* group, the CF effect leads to a higher incremental change have in output across all crop classes in tropical regions.

Globally, without CF, the most affected crops are *wheat*, *rice* and *oil seeds* (Table 4) with decreases in output for both irrigated and rainfed production in the case of *rice* and *wheat* (Table 5). The highest decreases in production volumes are obtained in India (−17% for *wheat* and −15% for *rice*), China (−11% for *rice*), Southeast Asia (−22% for *rice*) and Central Africa (−21% for *rice*). Except for *cane & beet*, the regional production levels of the other crop classes are also generally affected negatively. Gains in output are obtained in only a few cases and with a marginal contribution to the world crop output – Northeast Asia for all crop types, China for *wheat* (+1%), Northern Europe for *rice* (+34%), *other grains* (+2%), *veg & fruits* (+5%) and *oil seeds* (27%), North Latin America for *other grains* (14%), Northern Africa for *other grains* (+27%).

**Table 4:** Changes in crop output by crop type and by CO_2_ fertilisation variant – RCP 8.5.

Region	Wheat		Rice		Other grains		Veg & fruits		Fiber plants		Cane & beet		Oil seeds		Other crops	
	CF	w/o CF	CF	w/o CF	CF	w/o CF	CF	w/o CF	CF	w/o CF	CF	w/o CF	CF	w/o CF	CF	w/o CF
Australia & NZ	4%	−4%	8%	−17%	7%	−4%	2%	−5%	4%	−6%	30%	18%	8%	−8%	13%	−8%
Brazil	1%	−8%	3%	−12%	1%	−4%	2%	−6%	2%	−8%	13%	6%	4%	−20%	3%	−5%
Sahel	12%	−42%	8%	−21%	−2%	−7%	2%	−6%	6%	−10%	−3%	−11%	4%	−8%	3%	−26%
Central Africa	−6%	−25%	1%	−21%	−1%	−6%	1%	−11%	6%	−11%	43%	28%	5%	−19%	7%	−11%
Central Asia	4%	−6%	16%	−13%	4%	−2%	8%	−7%	10%	−12%	31%	1%	26%	−21%	34%	−12%
China	10%	1%	13%	−11%	−4%	−8%	9%	−7%	27%	−14%	32%	28%	20%	−2%	17%	−1%
Eurasia	3%	−7%	12%	2%	2%	−3%	3%	−8%	−9%	5%	3%	−7%	9%	−7%	10%	0%
India	4%	−17%	16%	−15%	−6%	−6%	11%	−24%	31%	12%	4%	4%	13%	−28%	7%	−9%
Middle East	0%	−7%	3%	−8%	0%	−7%	3%	−5%	3%	−3%	4%	−13%	6%	2%	5%	−11%
Northern Africa	−4%	−5%	8%	−6%	29%	27%	0%	−6%	−15%	−26%	13%	9%	1%	−3%	−1%	−6%
Northeast Asia	1%	10%	9%	−4%	14%	10%	3%	−1%	3%	13%	4%	2%	24%	21%	5%	0%
Northern Europe	4%	−3%	10%	34%	6%	2%	2%	5%	6%	−3%	7%	−2%	19%	27%	7%	3%
North Latin Am	4%	−18%	13%	−12%	16%	14%	4%	−11%	−3%	−3%	11%	4%	11%	−34%	8%	−8%
Canada	5%	−5%	4%	1%	3%	−5%	−1%	2%	−2%	1%	11%	−9%	6%	−16%	6%	−8%
Southern Africa	−1%	−9%	5%	−12%	2%	−5%	3%	−2%	5%	−9%	21%	16%	3%	−18%	10%	−3%
South Asia	3%	−22%	10%	−15%	−4%	−6%	1%	−3%	−9%	−12%	−10%	−15%	11%	−14%	−6%	−13%
Southeast Asia	−2%	−26%	9%	−22%	−14%	−13%	2%	−10%	2%	−7%	−6%	−8%	15%	−32%	2%	−14%
Southern Europe	−2%	−7%	4%	−5%	−1%	−9%	0%	−3%	6%	−3%	1%	−8%	2%	−4%	0%	−6%
South Latin Am	8%	−4%	18%	−6%	1%	−7%	4%	−10%	2%	-2%	42%	37%	7%	−17%	21%	3%
USA	4%	−4%	5%	−8%	0%	−12%	1%	−3%	3%	−9%	15%	−1%	6%	−14%	6%	−10%
Mean	2.6%	−10.4%	8.7%	−8.6%	2.7%	−2.6%	3.0%	−5.9%	4.0%	−5.4%	13.2%	4.0%	10.0%	−10.9%	8.0%	−7.3%
σ	4.2%	11.3%	4.7%	11.8%	8.8%	9.2%	2.8%	5.9%	10.2%	8.6%	14.9%	14.4%	7.2%	15.1%	8.4%	6.5%

Source: own calculation.

**Table 5:** Changes in regional crop production by crop type and by growing method – RCP 8.5 without CO_2_ fertilisation.

Region	Wheat		Rice		Other grains		Veg & fruits		Fiber plants		Cane & beet		Oil seeds		Other crops	
	Irrigated	Rainfed	Irrigated	Rainfed	Irrigated	Rainfed	Irrigated	Rainfed	Irrigated	Rainfed	Irrigated	Rainfed	Irrigated	Rainfed	Irrigated	Rainfed
Australia & NZ	−19%	−4%	−13%	−50%	2%	−4%	−7%	−7%	5%	0%	35%	−30%	97%	−12%	19%	−20%
Brazil	−8%	−10%	−11%	−14%	−12%	−3%	2%	−9%	12%	−8%	−12%	6%	20%	−23%	1%	−9%
Sahel	−32%	−4%	−16%	2%	13%	−5%	5%	−6%	46%	62%	−13%	−9%	−24%	−16%	−19%	−7%
Central Africa	−65%	−24%	36%	−33%	89%	−7%	−7%	−13%	−43%	−6%	−63%	32%	74%	−20%	−12%	−11%
Central Asia	3%	−12%	−15%	34%	−3%	−4%	−18%	23%	12%	−27%	2%	−31%	16%	−49%	−16%	−25%
China	−10%	18%	−10%	−9%	11%	−17%	−1%	−14%	52%	−17%	−11%	61%	26%	−24%	−4%	−3%
Eurasia	−14%	−5%	−33%	34%	0%	−2%	4%	−9%	24%	−7%	−9%	−5%	−10%	−7%	−24%	3%
India	−16%	−37%	−8%	−85%	−9%	−7%	22%	−51%	−51%	48%	−61%	586%	48%	−31%	−29%	−4%
Middle East	−12%	−3%	−6%	102%	0%	−7%	−2%	−13%	18%	45%	−10%	−25%	−5%	15%	−12%	−9%
Northern Africa	−34%	−10%	−21%	−4%	20%	13%	−2%	−23%	8%	28%	0%	5%	212%	−54%	−6%	−7%
Northeast Asia	10%	13%	0%	−28%	14%	14%	−3%	−9%	10%	15%	7%	−4%	41%	24%	12%	−5%
Northern Europe	−21%	−2%	−3%	−91%	−51%	45%	−3%	−18%	−21%	−13%	12%	−46%	−2%	−2%	−3%	−15%
North Latin Am	−13%	−2%	88%	30%	9%	2%	9%	2%	6%	32%	7%	−3%	−26%	28%	13%	2%
Canada	−42%	−35%	15%	−52%	−9%	−5%	−16%	−6%	24%	−6%	−33%	41%	4%	−60%	−3%	−34%
Southern Africa	−20%	−53%	−14%	−89%	−4%	−22%	−3%	−11%	−11%	−12%	−28%	1303%	12%	−26%	−13%	−4%
South Asia	−20%	−1%	−18%	69%	51%	−23%	−10%	−10%	−11%	7%	24%	90%	52%	−26%	−3%	7%
Southeast Asia	−22%	−29%	−18%	−27%	−21%	−12%	6%	−17%	−4%	−3%	−33%	44%	−37%	−32%	−5%	−29%
Southern Europe	−27%	−9%	−5%	−2%	119%	−14%	−2%	64%	−15%	38%	26%	10%	60%	−21%	−7%	2%
South Latin Am	−9%	−7%	−6%	−7%	7%	−11%	2%	−9%	19%	−51%	−2%	−12%	−9%	−2%	5%	−18%
USA	−25%	2%	−7%	−30%	−14%	−11%	−3%	−4%	4%	−17%	−5%	3%	−7%	−17%	−9%	−10%
**World**	−**15%**	−**4%**	−**8%**	−**26%**	**5%**	−**6%**	−**1%**	−**14%**	**1%**	−**2%**	−**17%**	**21%**	**9%**	−**20%**	−**3%**	−**9%**

Source: own calculation.

When embedding the CF effect, crop production generally increases with the highest impacts in China for *wheat* (+10%), rice (+13%), *veg & fruits* (+9*%), fiber plants* (+27%) and *oil seeds* (+20%), in India for *rice* (+16%) and *veg & fruits* (+11%), Northeast Asia for *rice* (+9%), Southeast Asia for *rice* (+9%) and *oil seeds* (+15%). At the same time, output decreases in a few instances due to a re-allocation of agricultural land to more productive crops – Northern Africa for *fiber plants*, Central Asia and China for *other grains,* Southern Europe for *wheat*.

The changes of export levels corresponding to the alterations to crop production patterns (Table 6). For RCP 8.5 without CF, exports of *wheat* decrease across all regions in the range of 2–8% from baseline values in 2050. For the other crop classes, some regions expand exports given the relative changes of yields and consequently the regional comparative advantage. In absolute traded volumes, the most significant increases in exports occur in Southeast Asia for *rice*, *other grains*, *cane & beet* and *oil seeds*, in China for *rice*, in South Asia for *veg & fruits* and *cane & beet*, and in Sahel and Central Africa for *veg & fruits*.In line with the overall increase in crop output, CF generally plays a positive role for exports with only a few cases of a decline induced by a loss of comparative advantage – *rice* in Australia & NZ, *other grains* in Northern Africa, *veg & fruits* in Sahel, Central Africa, Northeast Asia and Canada, *cane & beet* in Northern Africa. As already suggested in Fig. 3, the variance between regions in both output and export terms is lower when CF is embedded indicating that the addition of the fertilisation effect narrows down the differences in prices competitiveness and output levels across crops and regions.

**Table 6:** Changes in crop exports by crop type and by CO_2_ fertilisation variant – RCP 8.5.

Region	Wheat		Rice		Other grains		Veg & fruits		Fiber plants		Cane & beet		Oil seeds		Other crops	
	CF	w/o CF	CF	w/o CF	CF	w/o CF	CF	w/o CF	CF	w/o CF	CF	w/o CF	CF	w/o CF	CF	w/o CF
Australia & NZ	4%	−3%	−1%	−22%	7%	−4%	0%	−8%	7%	−10%	27%	9%	4%	−5%	7%	−10%
Brazil	8%	−4%	3%	−4%	2%	−4%	3%	−9%	7%	−12%	2%	−2%	5%	−21%	8%	−11%
Sahel	9%	−8%	7%	16%	6%	−3%	−4%	9%	6%	−10%	18%	5%	7%	−19%	7%	−3%
Central Africa	5%	−2%	10%	10%	5%	−4%	−3%	7%	3%	−11%	17%	5%	6%	−9%	9%	−11%
Central Asia	8%	−8%	7%	−41%	1%	−3%	7%	−9%	9%	−12%	30%	1%	7%	−20%	4%	−8%
China	5%	−3%	11%	10%	5%	−3%	2%	−5%	5%	−11%	12%	1%	7%	−19%	7%	−6%
Eurasia	7%	−4%	7%	−18%	1%	−4%	2%	−8%	19%	−13%	14%	17%	8%	−19%	6%	−9%
India	5%	−2%	12%	−26%	8%	3%	5%	−5%	9%	−8%	4%	−1%	5%	−15%	8%	−7%
Middle East	6%	−3%	7%	−3%	5%	1%	1%	−6%	11%	−10%	13%	3%	3%	15%	8%	−10%
Northern Africa	3%	−5%	8%	−15%	−3%	−13%	5%	−9%	1%	−20%	−11%	−12%	8%	−18%	9%	−8%
Northeast Asia	3%	−5%	5%	24%	7%	−1%	−1%	−22%	15%	−9%	12%	3%	2%	11%	5%	−12%
Northern Europe	4%	−7%	14%	−28%	1%	−7%	3%	−7%	12%	−12%	9%	−2%	6%	−22%	10%	−14%
North Latin Am	5%	−5%	1%	−15%	−3%	−15%	5%	−13%	3%	−11%	9%	0%	8%	−23%	9%	−12%
Canada	4%	−5%	15%	−13%	4%	−5%	−1%	4%	−8%	−22%	8%	5%	5%	−15%	6%	−12%
Southern Africa	9%	−6%	12%	−10%	7%	−2%	3%	5%	7%	−10%	13%	1%	8%	−11%	5%	−9%
South Asia	4%	−4%	11%	−21%	5%	−6%	−3%	7%	10%	−9%	18%	6%	5%	−11%	3%	−9%
Southeast Asia	5%	−2%	13%	20%	9%	2%	−2%	−5%	9%	−12%	24%	9%	1%	10%	6%	−9%
Southern Europe	5%	−5%	13%	−27%	0%	−8%	3%	−8%	17%	−4%	10%	0%	7%	−26%	9%	−9%
South Latin Am	8%	−3%	33%	−7%	−2%	−−9%	3%	−11%	5%	−12%	18%	5%	4%	−16%	4%	−11%
USA	4%	−4%	4%	−3%	−3%	−15%	0%	−2%	3%	−10%	27%	10%	4%	−17%	6%	−10%
Mean	5.6%	−4.4%	9.7%	−8.5%	3.1%	−5.0%	1.3%	−4.7%	7.6%	−11.3%	13.7%	3.1%	5.4%	−12.5%	6.8%	−9.5%
σ	1.9%	1.8%	6.9%	17.2%	3.8%	5.0%	3.0%	7.5%	5.8%	3.7%	9.3%	5.8%	2.0%	11.4%	1.9%	2.2%

Source: own calculation.

### 3.5 Decomposition of regional water CWP changes

Regional crop water productivities (CWP) calculated as the ratio between total irrigated crop outputs to regional irrigation water requirements. Changes in CWP can be explained through the three main drivers: (1) water re-allocation across crop types through differentiated yield changes (endogenous), (2) changes in natural soil moisture of irrigated land (exogenous) and (3) fertilisation water efficiency gains from evapotranspiration (exogenous). The endogenous/exogenous distinction is made based on whether the driver determines or not a change to the RESCU-Water model solution and implicitly on whether it affects crop output and irrigation allocation.

The effects are calculated as follows and comprise the responses at the three levels considered:

• *Yield changes* (all three response levels)– changes in water requirements relative to the baseline due to climate change impacts on yields but without changing the baseline water intensities
• *Soil moisture* (climatic and crop-level responses) – additional changes in water requirements by updating the water intensities to the “w/o CF” scenarios values. These reflect the changes in natural soil water balances when factoring in changes in climatic conditions
• *CF water efficiency* (climatic and crop-level responses) – additional changes in water requirements by further updating water intensities to “CF” scenarios values.

**Fig. 6:**
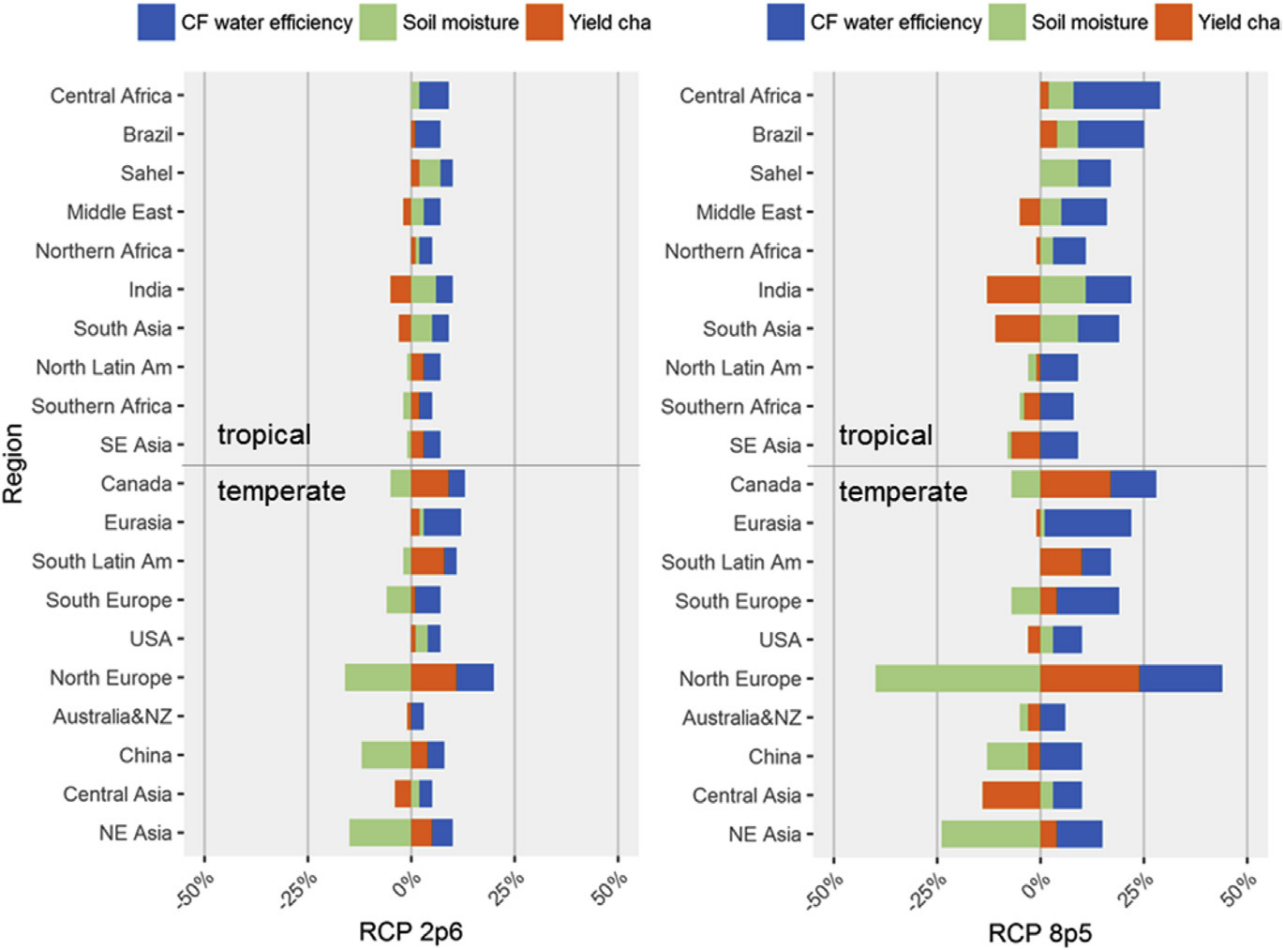
Decomposition of water productivity changes in scenarios with CO_2_ fertilisation. Source: own construction.

With CF embedded, CWP is higher than in the baseline in all tropical regions, whereas the outcome is mixed for temperate areas as China and NE Asia continue to be negatively affected in both RCPs and Central Asia in RCP8.5. The water efficiency gains induced by CF increases CWP in all regions (Fig. 6) and has the strongest impact among the three drivers in most cases. Hence this effect determines many regions to switch from a decline in water productivity to an increase, among which India and USA – regions which account for an important share in world irrigation withdrawals.

A contrast emerges between tropical and temperate regions in which the impact of soil moisture over CWP is significant. Tropical areas generally benefit from higher soil moisture requiring less irrigation water to compensate for soil water deficiencies. Nevertheless, this positive impact is entirely or partially offset in water-stressed regions (India, South Asia, Middle East, Northern Africa) through irrigation re-allocations to more water-intensive crops due to relative yield changes.

Another important observation is that the effects of CF water efficiency and soil moisture over CWP increase with CO_2_ concentrations. This amplification is also generally applicable to yield changes, except China, Eurasia, Southern Africa, USA and North Latin America where the sign of yield impacts shifts from positive to negative when moving from RCP2.6 to RCP8.5.

## 4 Discussion

The results in this study indicate that changes in climatic conditions (temperature and precipitation) will negatively impact crop production by 2050 in all but a few instances (sugarcane in general and specific crops in a few regions) even in the low-emissions scenario RCP2.6. The cases for which an increase in production was obtained only lead to a marginal effect on world crop output. Therefore, the results indicate a weak re-location of production across the globe with a reduction in exports in most instances.

The addition of the CF effect on yields and irrigation water intensities has a strong offsetting impact on crop output and irrigation water requirements leading to a positive outcome across the board compared to the 2004–2050 baseline – higher crop output, lower crop prices, lower irrigation withdrawals and smaller differences across regions. The crop water efficiency due to CF also plays a dominant role in CWP increases, especially in tropical regions.

The obtained changes in irrigation water requirements and CWP for the two CF variants are only partially explained by yield alterations, whereas changes in soil moisture and CF-induced water efficiency gains are also significant and even have a larger effect in many regions. Therefore, the inclusion of irrigation water intensity as a parameter influenced by climate change is an important addition to the current economic assessments of climate change incidence on crop output and water demand in agriculture, as previous efforts have mostly focused on changes in yields alone.

The adverse effects of changes in climatic conditions on crop output could be attenuated through several adaptation measures (Porter et al., 2014). One measure captured in the model simulations is the inter-crop substitution given relative yield changes. However, this could lead to an undesirable reduction in the production of staple crops. Other crop management measures which are not considered but which could have a significant impact on yields include changes in sowing dates to cooler months and the use of cultivars tolerant to high temperatures. However, at this stage, the implications of these solutions on water demand are not clear for extended geographical areas.

The study also addresses the uncertainty of climate change incidence over crop production with respect to climatic conditions through the use of multiple GCMs. This approach could be extended to crop performance uncertainty by using yield and irrigation water intensity data from alternative crop models. Although ISI-MIP publishes results from other global models, there is a wide variation in the coverage of results in terms crop classes, CF variants and RCPs. Data from all other models, at the time of this analysis, does not cover all the relevant cases considered here. Due to the significant impact of CF on crop water productivity, the inclusion of further crop modelling data incorporating insights from new field experiments would be welcome.

Compared to the yield impacts implemented in Nelson et al. (2014), the incidence of climate change is considered in higher detail both through the inclusion of a larger number of crop types^3^ and through the differentiated specification of rainfed and irrigated production. This wider coverage enables a more complete assessment of changes in crop production and prices, and the corresponding implications for crop availability. The results in this analysis show a marked contrast between the negative impacts over *wheat*, *rice* and *oil seeds* and the positive impacts over *cane & beet*, indicating significant future alterations to the crop production mix and nutrition coming from changes in climatic conditions. Thus, regions representing a large share of the world population (India, China, Southeast Asia and Central Africa) could face decreases of 10% or more in basic crops production with important implications for food security.

While there is a good accordance with the changes in yields and water intensities from Konzmann et al. (2013) across both CF variants, the water requirements calculated with LPJmL crop modelling framework alone without the output response from an economic model leads to significantly different results – an increase in global withdrawals of over 20% by 2080 in the ‘without CF’ scenario for SRES A2 (comparable to RCP8.5). The use of an economy-wide model (RESCU-Water) to determine changes in crop output captures the re-allocation of means of production across crops given relative yield changes between crop types and growing methods. This advanced specification allows for a more accurate calculation of the resulting changes in irrigation water requirements following the substitution effects between irrigated and rainfed production driven by market prices – a determinant also left out in Fischer et al. (2007) which determine a 45% increase in global withdrawals due to changes in climatic conditions in the 2000–2080 horizon for SRES A2r.

Compared to Nelson et al. (2009) which use a partial-equilibrium model (IMPACT) with the inclusion of water availability constraints in addition to yield changes for irrigated and rainfed crops, the determined changes in crop output for 2050 without CF are consistent in sign but of a lower magnitude e.g. a strong reduction in *rice* output in Southern and Eastern Asia, a positive impact on *wheat* output in Eastern Asia, negative impact on other grains in *Sub-Saharan Africa*. Hence, the addition of water availability considerations could further impact crop output notably in regions for which CWP is negatively affected by a reduction in soil moisture as determined in Section 3.5.

One limitation of the crop modelling data used in this study is that the effects associated with CF strictly refer to current crop management conditions and are taken in isolation from the interactions with other GHG types. Therefore, the yield and water intensities values employed in the model scenarios do not embed the damaging effect of ozone over crop photosynthesis (see, for instance, McGrath et al., 2015). With the current emission patterns, the rise in CO_2_ emissions will also be accompanied by increases in ozone precursor concentrations, leading to a non-linear impact of CF over crop performance (Porter et al., 2014). Yield could also be impacted by a likely decrease in herbicide effectiveness with the increase in CO_2_ concentrations.

Another limitation applicable to both CF variants is that the impacts considered in the model simulations are a reflection of changes in conditions on the land areas currently harvested. With climate change, areas less used presently for crop production could become suitable in the future, leading to a positive impact on yields mainly in the high-latitude regions (IPCC, 2014). This could further increase the differences in climate change incidence between temperate and tropical areas from those obtained in this study.

The multi-model approach to cover the climate-crop-economic uncertainty dimensions undertaken in Nelson et al. (2014) to include future water requirements would also require the comparison of the RESCU-Water model results with other economic models. To the author's best knowledge, the expansion of irrigation as a function of market forces enabling regional changes to irrigation water requirements is possible only in a few water-focused models^4^ and was undertaken only in one instance (Fischer et al., 2007). Therefore, more economic modelling work would be needed in order to have a similar approach to that for land-use responses in Nelson et al. (2014) but applied to blue water in crop production.

The socioeconomic and technological evolution of the SSP2 storyline is considered to be incompatible with RCP2.6 (O'Neill et al., 2014). Therefore, the drastic reduction in emissions for RCP2.6 which would limit the global mean temperature increase to below 2 °C could require a large-scale adoption of bioenergy. A few global studies have considered the relationship between bioenergy-based mitigation strategies, land use (Lotze-Campen et al., 2014) and food security (Hasegawa et al., 2018). However, considering the currently low penetration of purpose-grown crops (e.g. switchgrass), there is no agreement on how to capture the effect of their cultivation on the competition for land with other use types (food crops, forestry, pasture or unmanaged land). Furthermore, the implications of second-generation crops on irrigation water demand are unclear given that many analyses start from the assumption that these can grow without the use of irrigation (e.g. Harto et al., 2010). The bioenergy potential and the resulting irrigation requirements will be dependent on crop management practices (nutrient and water input, crop rotation and multi-cropping) (Kang et al., 2014; Li et al., 2018) and future land-use policies. Therefore, the additional implications of bioenergy production on crop output and water requirements are left for further dedicated assessments which would need to address the implied uncertainties.

## 5 Conclusions

The analysis in this article determined the impacts of climate change on irrigation water requirements explained through biophysical alterations to irrigation water intensities coming from soil moisture and evapotranspiration changes, but also through alterations to the global patterns of crop production induced by economic responses to yield changes. As already highlighted in the IPCC literature, higher CO_2_ atmospheric concentrations determine very different impacts on crop production depending on whether CF is taken into account. The contrasts between the two CF variants in terms of changes to regional crop output and irrigation requirements, and the variance of impacts between regions increases with CO_2_ concentrations.

Changes in climatic conditions without the CF effect lead to an overall decrease crop output driving down irrigation water requirements – contrary to results found in other studies. The negative impacts on crop production and CWP are more pronounced for tropical than for temperate regions. Furthermore, adaptation to climate change mostly takes place through inter-crop substitution and less through a significant shift to irrigated production. The crop production mix could thus be altered towards more sugarcane output in the detriment of rice, wheat and oils seeds.

A different outcome is obtained when the CF effect is included. Regional output generally increases across all crop classes, leading to a more balanced regional production of grains, oils and sugars, whilst crop output and price impact disparities between regions are also becoming narrower. Water requirements are considerably lower than in the baseline given the overall boost in water productivity induced by CF water efficiency gains. This reduction in water demand from crop production could free up important water resources for other uses.

Considering the significant impact of CF over crop output and water resources, more work is welcome in order to reduce the uncertainty of this dimension in the crop growing conditions. At the same time, a comparison of biophysical changes obtained through multiple crop models would be desirable for an increased diversity in modelling of yield and crop water efficiency responses.

## Declarations

### Author contribution statement

Victor Nechifor: Conceived and designed the experiments; Performed the experiments; Analyzed and interpreted the data; Contributed reagents, materials, analysis tools or data; Wrote the paper.

Matthew Winning: Contributed reagents, materials, analysis tools or data.

### Funding statement

Victor Nechifor was supported by FutureDAMS – (ESRC ES/P011373/1).

### Competing interest statement

The authors declare no conflict of interest.

### Additional information

No additional information is available for this paper.

## Footnotes

1 C3 and C4 plants use different processes for carbon fixation through photosynthesis with C3 plants having a lower CO_2_ absorption efficiency. Whilst most plants belong to the C3 category, the most important examples of C4 crops are maize and sugarcane.

2 https://esg.pik-potsdam.de/search/isimip-ft/.

3 Nelson et al. (2014) account for yield changes only for rice, maize, coarse grains and oil seeds.

4 Partial-equilibrium IMPACT and MAgPIE models; CGE EPPA and BLS-AEZ models.
